# Stewardship 2.0: Embracing elements of implementation science to enhance everyday antimicrobial Stewardship efforts

**DOI:** 10.1017/ash.2023.413

**Published:** 2023-09-07

**Authors:** Elizabeth Monsees, Ann L. Wirtz, Sarah Krein

**Affiliations:** 1 Performance Excellence, Children’s Mercy Hospital, Kansas City, MO, USA; 2 School of Medicine, University of Missouri, Kansas City, MO, USA; 3 Pharmacy, Children’s Mercy Hospital, Kansas City, MO, USA; 4 Center for Clinical Management Research, VA Ann Arbor Healthcare System, Ann Arbor, MI, USA; 5 Department of Internal Medicine, Division of General Medicine, University of Michigan Medical School, Ann Arbor, MI, USA

**Keywords:** Implementation science, quality improvement, antimicrobial stewardship

## Abstract

This article explores the utility of implementation science (IS) as a method to promote the effective uptake of antimicrobial stewardship processes. Elements of IS can be readily incorporated into QI work and used as a platform to extend stewardship reach. As stewards are stretched to do more, IS can be a potential vehicle to ensure that our collective work is impactful, sustainable, and contributes more broadly to clinically relevant improvements.

The role of antimicrobial stewardship (AS) is rapidly expanding, with stewardship activities crossing into different venues (i.e., ambulatory settings) and domains (e.g., diagnostic stewardship, pandemic stewardship, personal protection equipment stewardship, vaccine stewardship).^1,2^ In addition, revised standards issued by The Joint Commission, effective January 1, 2023, increased expectations for antibiotic stewardship programs (ASP) in the hospital setting.^3^ This includes new implementation and evaluation-focused quality improvement (QI) requirements related to antibiotic use. Shifting into this new era of stewardship 2.0 requires new approaches to extend the daily work of stewards and stewardship reach. Specifically, within the QI arena, we believe that implementation science (IS) is a potential vehicle to ensure our collective work is impactful, sustainable, and applicable to others outside of our institution.

## IS and quality improvement, two peas in a pod?

Stewards are familiar with QI, which is commonly utilized to improve antibiotic prescribing and measure intervention outcomes.^4^ Quality improvement focuses on improving local processes and structures to obtain desirable results, with quick tests of change to determine intervention effectiveness.^5,6^ For example, at Children’s Mercy Hospital our ASP has several ongoing QI projects to address local issues, such as improving penicillin allergy documentation at an urgent care site or implementing an algorithm for blood culture collection processes in the intensive care unit. Both are designed to address local issues, involve iterative tests of change, and are intended to improve care within a single institution.

Increasingly multiple scientific journals, including *Antimicrobial Stewardship & Healthcare Epidemiology*, have promoted the use of IS to guide the development and use of impactful and sustainable AS interventions. IS is defined as “the study of methods to promote the systematic uptake of research findings and other evidence-based practices into routine practice and, hence, to improve the quality and effectiveness of health services.”^7^ In contrast to QI, which is solution-focused and internally driven, drawing largely on principles and tools for improving industrial processes, IS focuses on producing generalizable knowledge and promoting the systematic use of effective practices, using principles and tools from the behavioral and social sciences.^5,8,9^ As Livorsi and colleagues underscore in their Society for Healthcare Epidemiology of America White Paper, IS gives direction or strategy to facilitate clinician adoption and the accompanying actionable frameworks to manage implementation processes.^10^ The authors also identify different implementation strategies (evaluative and iterative strategies, develop stakeholder relationships, train and educate stakeholders, support clinicians, change infrastructure, adapt and tailor to the context, provider interactive assistance, engage consumers, and utilize financial strategies), IS frameworks (process, determinant, evaluation), and IS outcomes (acceptability, adaption, adoption, appropriateness, cost, feasibility, fidelity, penetration, and sustainability).^10^

Nonetheless, with over 80 available frameworks, IS can understandably seem overwhelming and confusing for the everyday steward. Unbeknownst to many stewards, aspects of daily AS work often contain elements of IS. For example, modifying order sets or developing clinical practice guidelines are types of implementation strategies to improve antibiotic prescribing. Stewards also often informally consider internal and external barriers that may influence uptake of an antibiotic use protocol or guideline. In addition, opportunities where QI and IS can be mutually beneficial are increasingly being identified.^5,8,11,12^ The Quality Enhancement Research Initiative (QUERI) Implementation Roadmap, for example, is a resource aimed at researchers and implementation practitioners, such as stewards, and incorporates practical approaches to guide implementation and QI efforts.^13^ Given this symbiotic relationship between IS and QI, we propose a simple approach to facilitate incorporating IS into QI work.

**Figure 1.**
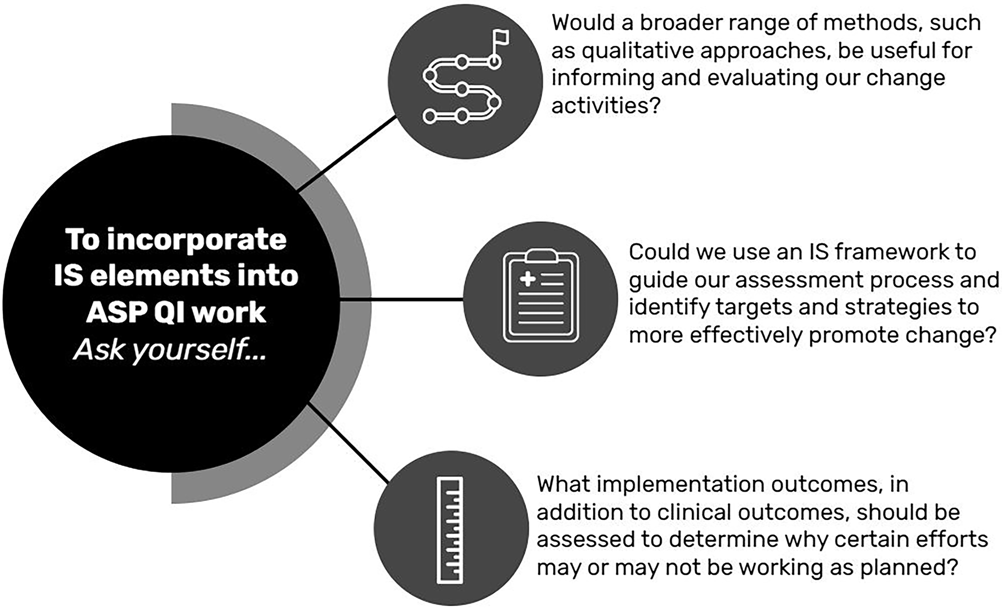
Opportunities for Implementation Science in ASP QI Projects.

Specifically, we believe stewards can incorporate elements of IS by asking themselves these three questions during QI project planning: (1) Would a broader range of methods, such as qualitative approaches, be useful for informing and evaluating our change activities?; (2) Could we use an IS framework to guide our assessment process and identify targets and strategies to more effectively promote change?; and (3) What implementation outcomes, in addition to clinical outcomes, should be assessed to determine why certain efforts may or may not be working as planned? (Figure 1)

## Real-life application, how can we apply IS elements to our own work?

Below we describe an example of a QI project conducted at Children’s Mercy Hospital, and we retrospectively reflect on how integration of additional IS elements may have enhanced our efforts.

In 2019, nurses working on a surgical floor recognized that a large proportion of post-operative cefazolin doses prepared for patients were being wasted. Doses were returned to the pharmacy unused at patient discharge for various reasons. Upon initial review of pharmacy billing data from 2018, ASP identified that 18,501 doses of antimicrobials were wasted, which translated to approximately $252,810 in drug cost alone. Cefazolin accounted for 15% of waste, and the nursing unit who identified the problem was a top contributor.

To investigate reasons for cefazolin waste, nurses completed an abnormality tracker for wasted doses and ASP performed weekly reviews of the medical record for patients with wasted doses. The majority of wasted cefazolin doses were from surgical services. Doses were commonly wasted due to patient discharge as post-operative antibiotics were often ordered for a longer duration than patient length of stay (e.g., cefazolin ordered for 7 days and patient was hospitalized for 2 days). Pharmacy prepares doses several hours prior to administration; therefore, previously prepared doses were wasted upon discharge. Multiple post-operative doses were also wasted due to a timing issue within the electronic medical record (EMR) which prevented pharmacists from appropriately retiming the post-operative dose at the point of order verification. This frequently resulted in duplicate doses being dispensed.

Our team formed a group of stakeholders including nurses, pharmacists, QI consultants, and an anesthesiologist to identify intervention strategies to reduce post-operative cefazolin waste based on ease and perceived impact. The first intervention included the addition of default antibiotic durations for post-operative cefazolin orders on surgical order sets for the Plastic Surgery service. Despite the duration adjustment, this did not result in a significant decrease in wasted post-operative cefazolin doses. The next intervention included the addition of a time extension to post-operative antibiotic orders on order sets that mitigated the EMR issue. This allowed pharmacists to appropriately schedule the first post-operative cefazolin dose at the appropriate interval following the last intra-operative dose. Education was provided to pharmacists verifying these orders. This intervention resulted in a 16% decrease in the odds of wasted post-operative cefazolin when comparing the time periods before and after implementation. While this was impactful, ASP continues to investigate reasons for cefazolin post-operative waste as it is still a common occurrence.

This QI project was beneficial at our institution, but elements of IS could have potentially enhanced its effect through incorporation of the questions outlined in Figure 1. Overall, using qualitative methods to gain meaningful insights and to establish key stakeholder relationships with nursing, floor pharmacists, and anesthesia was helpful for ASP to understand issues with antibiotic timing and verification. However, as outlined in question two, this could have been enriched with the use of IS frameworks, such as the Consolidated Framework for Implementation Research and a brief pragmatic assessment to systematically assess contextual factors that can influence our planned practice change, which in turn might help identify potential implementation strategies.^14^ For example, the use of evaluative and iterative strategies such as audit and feedback may have been effective in providing real-time results to surgeons. Finally, with question three, measurement of certain IS outcomes also may have enhanced our assessment and understanding of our results. Acceptability measurements would have determined whether Plastic Surgery was willing to use the shorter, defaulted durations of antibiotics. Fidelity assessments would have verified whether pharmacists were appropriately retiming the post-operative cefazolin doses following the time extension in the EMR.

As demonstrated in our cefazolin example, IS can provide a systematic approach to identifying potential barriers and facilitators to QI change interventions. This in turn can point to more effective implementation strategies, organizational and/or behavioral, to promote practice change. Moreover, while the improvement project was fruitful, and addressed a particularly unsettling issue to most stewards, wasted antibiotics, our knowledge of what made certain aspects of this project successful is limited. Evaluating implementation outcomes would have provided critical information related to intervention use (e.g., what works) and sustainability. These same principles could be applied to other improvement activities that stewards encounter on a day-to-day basis, such development of evidence-based guidelines outlined in the TJC standards.

In sum, the rigor provided by IS offers structure for common interventions and activities led by stewardship programs and can better inform strategies for broad-scale change. Budding implementation enthusiasts can begin incorporating IS into regular QI work by asking three simple questions that may help stewards discover what works best for the adoption of new practices by focusing on methodological approaches that *inform* change, IS frameworks that *promote* change, and outcome data that *sustain* change. As stewards are stretched to do more, IS can be a potential vehicle to ensure our collective work is impactful, sustainable, and contributes more broadly to clinically relevant improvements.
